# The Role of Emotional Intelligence in the Maintenance of Depression Symptoms and Loneliness Among Children

**DOI:** 10.3389/fpsyg.2019.01672

**Published:** 2019-07-17

**Authors:** Sarah K. Davis, Rebecca Nowland, Pamela Qualter

**Affiliations:** ^1^Department of Psychology, Institute of Health and Society, University of Worcester, Worcester, United Kingdom; ^2^Child Health and Well-Being Research Group, School of Nursing, University of Central Lancashire, Preston, United Kingdom; ^3^Manchester Institute of Education, The University of Manchester, Manchester, United Kingdom

**Keywords:** emotional intelligence, emotional self-efficacy, depression, loneliness, children, internalizing problems, social emotional learning, gender

## Abstract

Identifying factors that predict the maintenance of depression and loneliness in children is important for intervention design. Whilst emotional intelligence (EI) has been identified as a predictor of mental health, research examining how *both* trait and ability EI contribute to long-term patterns of symptomatology in children is markedly absent. We examined the impact of both TEI and AEI on the maintenance of loneliness and depressive symptoms over 1 year in children aged 9–11 years. Two hundred and thirteen children (54% male) completed the TEIQue-CF and the MSCEIT-YV at the first time point of the study, and the Child Depression Inventory and the Loneliness and Aloneness Scale for Children and Adolescents at Time 1 and, again, 1 year later. Findings indicate that emotional skills (AEI) are important for predicting the maintenance of depressive symptoms and loneliness in children over 1 year; emotional self-competency (TEI) is less influential, only contributing to long-term loneliness in girls. Moreover, whilst deficiencies in the ability to perceive and understand emotions were predictive of prolonged symptomatology, so, too, were *proficiencies* in using emotion to facilitate thinking and emotion management. Those findings carry important implications for EI theory and future research. They also indicate that EI interventions tailored to groups of “at risk” school children may be useful for reducing specific profiles of internalizing symptoms. Programs targeting AEI skills may be universally helpful for reducing the likelihood that depressive symptoms and loneliness will be maintained over time in middle childhood; girls at risk for prolonged loneliness would additionally benefit from opportunities to bolster TEI.

## Introduction

Developments in how we understand, use, and manage emotions during childhood are important for understanding mental health in later adolescence and adulthood (Jones et al., 2015). But, those skills are also crucial for understanding wellbeing, including mental health and social connection, during the childhood years. Internalizing problems among children have increased (Kieling et al., 2011; Husky et al., 2018) and feelings of loneliness are a particular issue, with 14% of children aged 10–12 reporting that they often feel lonely (Office for National Statistics [ONS], 2018a). It is therefore crucial to investigate whether individual differences in emotion capabilities can explain the maintenance of internalizing problems, including prolonged depression and loneliness (i.e., over a sustained period of time). In the current study, we explore the role of both emotional self-competency (Trait Emotional Intelligence; TEI) and emotional skills (Ability Emotional Intelligence; AEI) in the maintenance of loneliness and depressive symptoms over 1 year in a sample of children aged 9–11 years.

Although a recent meta-analysis suggested there has been no worsening of internalizing symptoms in the last decade among children and toddlers (Bor et al., 2014), when emotional distress among children is assessed through self-reports, a rise is evident (Husky et al., 2018). Thus it appears that once children have the cognitive capacity to self-report on their feelings, we are able to gain insight into the prevalence of depressive symptoms and feelings of loneliness that, by their very nature, may not be visible to external observers.

There is global concern for the mental health of young people and the scale of the problem calls for action (Kieling et al., 2011). UK data suggest that rates of mood disorders are increasing in children and adolescents, rising from 9.7% in 1999 to 11.2% in 2017 (Sadler et al., 2018). Studies have shown that in the general population 1.9–13.2% of boys and 1.2–26.0% of girls experience prolonged depressive symptoms (Wiesner and Kim, 2006; Dekker et al., 2007). If untreated, depressive symptoms during childhood and adolescence can lead to later impairments in adulthood (Costello et al., 2003; Belfer, 2008), impacting learning and academic performance (Vander Stoep et al., 2003) and eventual employment prospects (Veldman et al., 2015). Prevention science research suggests that key personal competencies (e.g., social and emotional competence) may play a protective role in the long-term maintenance of internalizing symptoms, including depressed mood (e.g., Obradović et al., 2010).

The number of people experiencing prolonged loneliness is also increasing (Victor et al., 2005; Gerst-Emerson and Jayawardhana, 2015), with a recent report stating that UK adolescents reported feeling lonely more often than adults (Office for National Statistics [ONS], 2018b). Loneliness among children in the UK is also highly prevalent (Office for National Statistics [ONS], 2018a). There has been little examination of the prevalence of enduring loneliness among children and adolescents, but where longitudinal studies have examined prolonged loneliness, prevalence varies from around 4.1–22% (Ladd and Ettekal, 2013; Qualter et al., 2013; Schinka et al., 2013), indicating that loneliness is a significant problem for some children. Loneliness is considered to be a normative experience that promotes reconnection with others (Qualter et al., 2015b), but when it is prolonged it impacts current well-being (Qualter et al., 2010; Eccles et al., in press), future physical health (Harris et al., 2013; Qualter et al., 2013), academic achievement (Benner, 2011), and increases in social withdrawal (Schinka et al., 2013). Thus, it is important to explore whether there are factors that prevent the maintenance of loneliness over time and promote reconnection.

Key developments in emotional competencies are apparent across childhood and early adolescence, and they are important for mental health in later adolescence and adulthood (Davis and Qualter, 2019). Young people who possess good levels of emotional knowledge to help them identify and recognize emotional cues in themselves and others, and who are able to effectively regulate internal emotional states, are more likely to experience success in navigating complex social interactions, reading and responding to others’ cues to establish and grow interpersonal relationships (Trentacosta and Fine, 2010). And, we would expect that youth with good knowledge of emotions are able to work out what they are feeling and why, making changes to their environment to ensure that negative affect does not become common-place or prolonged. Additionally, being adept at managing feelings, particularly negative affect, in a given situation should enable young people to deal with changes and challenges when they arise. Beliefs about emotional competencies, arising from prior experience, training etc., are also important for engaging in social interaction and effortful affect regulation (e.g., Tamir et al., 2007). Thus, having good emotional competencies may buffer children from the experience of prolonged internalizing behaviors (i.e., loneliness and depression).

In support of those ideas, early reports of emotional functioning (e.g., teacher reported emotion understanding and regulation) at 6 years of age have been found to predict internalizing problems (depressed, anxious, overcontrolled symptoms) 19 years later (Jones et al., 2015). Further, evidence shows that teaching emotion understanding and regulation as part of Social-Emotional Learning interventions is protective against the development of subsequent conduct problems and emotional distress, and useful for promoting social competency and pro-social attitudes (e.g., connectedness) (Taylor et al., 2017). Moreover, levels of emotional distress can be reduced among those reporting high levels at the start of interventions (Sklad et al., 2012; Domitrovich et al., 2017).

The field of emotional intelligence (EI) offers a useful organizing framework for examining the impact of emotional competencies on mental health in young people (Davis and Qualter, 2019). EI can be measured in two ways: either as a “trait” (TEI) tapping self-reported emotional competency (e.g., perceived emotional expressiveness) and personal qualities (e.g., assertiveness, empathic concern), or, as “ability” (AEI), through IQ-style testing of emotional skills (e.g., emotion perception, management etc.). AEI and TEI share differential patterns of association with broadband personality dimensions and cognitive ability and are often negligibly related (e.g., Davis and Humphrey, 2012). Rather than viewing AEI and TEI as competing, mutually exclusive perspectives on EI, many researchers now adopt the position that the two offer complementary approaches that shed important light on how maximal emotion knowledge, as well as typical emotional preferences, can influence everyday behaviors in meaningful ways (Mikolajczak, 2009).

Meta-analytic examination of emotion understanding in relation to mental health, using the AEI and TEI frameworks, has established that they both predict mental health among adults and adolescents aged over 15 years (Martins et al., 2010). That work suggested perceived emotional competency (TEI) was a stronger predictor of mental health than actual emotional skill (AEI), although both were negatively associated with a wide range of mental health problems. Recent work with children and adolescents supports those trends, with AEI and TEI being variously associated with a range of clinical symptoms including depression, anxiety, self-harm, suicidal ideation and attempts, disruptive behavior, and attention deficit hyperactivity disorder (Mavroveli et al., 2008; Cha and Nock, 2009; Mikolajczak et al., 2010; Rivers et al., 2012; Kristensen et al., 2014). Few studies compare the influence of perceived emotional competency (TEI) and skill (AEI) on symptoms, but where that comparison is made in cross-sectional studies, TEI has a stronger influence than AEI on internalizing symptoms in young people, and this relationship survives tests of shared conceptual overlap with broadband personality dimensions, criterion outcomes and common method variance (Williams et al., 2009, 2010; Davis and Humphrey, 2012). TEI can also predict depression, anxiety, and social stress in adolescents across a 1 to 2 year period (Salguero et al., 2012; Gomez-Baya et al., 2017). However, it remains to be established whether emotional skills and self-perceptions are also important for the maintenance of depression over time among pre-adolescence and childhood, something which is important given the developmental trajectories of the disorder and associated negative sequelae.

Trait Emotional Intelligence and AEI are also associated with behavior that increase positive affiliation with other people. For example, TEI and AEI are associated positively with prosocial behavior (Ciarrochi et al., 2000, 2001; Charbonneau and Nicol, 2002; Lopes et al., 2004, 2005; Mavroveli et al., 2009; Frederickson et al., 2012) and negatively with aggressive behavior (García-Sancho et al., 2016; Qualter et al., 2019). Such findings support the thesis that lower AEI and TEI are implicated in the maintenance of loneliness because the behavioral foundations for good quality relationships are not in place. Findings support that idea, showing that AEI is linked to social network size and quality of group and friendship interaction (Brackett et al., 2004; Lopes et al., 2004), and with loneliness (Zysberg, 2012). Prospective work shows that, more specifically, poor emotion regulation skills predict increases in loneliness over time in samples of young adults (Vanhalst et al., 2012; Nightingale et al., 2013) and young adolescents (Wols et al., 2015). Missing from the literature, however, is an examination of TEI and loneliness, and there are gaps in our understanding about whether emotion self-efficacies and skills together are implicated in the maintenance of loneliness among pre-adolescence.

Evidence suggests that, during adolescence, females are more affected by depression than males (e.g., Hankin et al., 1998), and males are lonelier than females (Koenig and Abrams, 1999). Gender has been shown to moderate the relationship between EI and wellbeing in some previous studies. For instance, adult females reported higher TEI than males (Salguero et al., 2012) whilst higher levels of AEI and depression were reported in young females compared to males (Davis and Humphrey, 2012). Further, a meta-analysis established that the relationship between TEI and mental health is stronger for females than males (Martins et al., 2010). The moderation of gender in the relationship between TEI and wellbeing is also evident in cross-sectional (Piqueras et al., 2019) and longitudinal work (Gomez-Baya et al., 2017), with the relationship between TEI (low levels of perceived clarity and repair; high levels of perceived attention to feelings) and depression over a two period more pronounced for adolescent females than males. Such evidence highlights the need to explore the moderating effects of gender in the relationship between AEI/TEI and maintenance of high levels of depressive symptoms and loneliness.

Empirical examination of factors that predict the maintenance of depression and loneliness is important because findings can influence the design of interventions, improving effectiveness. However, to date, most of the work linking EI and wellbeing uses cross-sectional designs with adolescent and adult groups, limiting its impact and significance to the debate regarding the maintenance of psychological conditions. Research examining how *both* TEI and AEI contribute to long-term patterns of symptomatology in children is markedly absent, despite cross-sectional data suggesting that emotional skill and perceived competency may differentially predict internalizing symptoms. Research designs where internalizing problems are monitored over time have the potential to offer clearer suggestions for interventions that will offer longer-term impacts on symptoms – this is pertinent to Social and emotional learning programs where the durability of intervention effects are yet to be established (e.g., Durlak et al., 2011). To address the gap in the extant literature for examination of the role of EI in the maintenance of internalizing problems, the current study explores the role of AEI skills and TEI in predicting the maintenance of high levels of loneliness and depressive symptoms in children over a 1-year period. We also examined whether that relationship was moderated by gender. Given the extant literature, we expected TEI to be a stronger predictor of prolonged depressive symptoms and prolonged loneliness than AEI. We also expected gender to moderate those effects.

## Materials and Methods

### Participants and Procedure

The participants were a sample of children from the Lancashire Longitudinal Study of Social and Emotional Development (LLSSED), which is a prospective study of 417 children recruited from schools in Lancashire, United Kingdom. Children provided self-reports of loneliness and depressive symptoms, at two waves that took place 12 months apart; they completed an AEI test and a TEI measure at the first wave. It took, on average, 1 h to complete the questionnaires and AEI test at Time 1, and 20 min to complete the questionnaires at Time 2. 213 children provided full sets of data at both time points. Mean age of the children in the current sample at the first measurement wave was 9 years and 3 months (SD = 6.2 months); 54% of the sample was male. The schools included in the study were representative of schools across the UK as determined by the government Index of Multiple Deprivation and eligibility of free school meals. The participant’s primary caregiver gave written, informed consent at each wave of data collection, and all participants provided verbal assent to take part in data collection. Participants were tested in accordance with the national and local ethics guidelines according to the Declaration of Helsinki. The Ethics Committee at University of Central Lancashire provided ethics approval for the study.

### Measures

#### The Child Depression Inventory (CDI; Kovacs, 1992)

The CDI was used to assess depressive symptoms. It is a 27-item scale, where each item consists of three choices (0, 1, and 2). Scores range from 0 to 20, with higher mean scores corresponding to higher depressive symptoms. An example item is “I do most things okay” (0), “I do many things wrong” (1), and “I do everything wrong” (2). The CDI scale has been found to display acceptable internal consistency, reliability, and validity (Kovacs and Beck, 1977; Kovacs, 1992). In the current study, the CDI demonstrated acceptable internal consistency at both time points [α = 82, and 80. at Time 1 (T1) and Time 2 (T2), respectively]. We used the clinical cut-off ≥12 for mild depression using CDI (Allgaier et al., 2012) to explore clinical levels of depression in the current sample. We found at T1 20 (9.4%) children in the current sample would be classified as having clinical levels of depression; at T2, that number was 35 (16.4%); 16 (7.5) children reported those clinical levels at both T1 to T2. Those percentages are slightly above that found in the Allgaier et al. (2012), paper, but fit with the latest UK statistics on mood disorders for children of this age (Sadler et al., 2018). When using a more conservative cut-off ≥20 (Rivera et al., 2005), we do not find any children at T1 or T2 who would be classified as clinically depressed. No parents or children reported receiving treatment for depression during the 12 months of the study.

#### Louvain Loneliness Scale for Children and Adolescents (LACA: Marcoen and Brumage, 1985)

Loneliness in relation to peers was measured using the peer sub-scale of the LACA. LACA is a 48 item scale, separated into 4 scales of 12 items The peer sub-scale was used in the present study and includes items “I feel isolated from other people” and “I feel excluded by my classmates.” Children are asked to indicate how often each item applies to them on a 4-point scale: 4 (often), 3 (sometimes), 2 (rarely), or 1 (never). Higher mean scores on the scale are indicative of greater loneliness in relation to peers. The LACA has been found to display acceptable internal consistency, reliability, and validity (Maes et al., 2015a,b). Although originally used with Dutch-speaking children it has also been used with English-speaking children (Terrell-Deutsch, 1999; De Roiste, 2000; Qualter and Munn, 2002, 2005; Harris et al., 2013; Qualter et al., 2013). In the current study, this sub-scale demonstrated acceptable internal consistency across the two waves of data collection (α = 79 and 0.82. at T1 and T2, respectively). There are no clinical cut-off points for loneliness, but to explore how many children were scoring at the higher end of loneliness, we explored how many children answered “often” to more than half the questions at T1 and T2. Findings showed that 22 children (10.3%) at T1 and 41 children (19.2%) at T2 reported feeling lonely often; 21 children (9.9%) reported those high levels at both T1 and T2.

#### Mayer-Salovey-Caruso Emotional Intelligence Test-Youth Version (MSCEIT-Yv; Mayer et al., 2005)

The scale consists of 101 items (of which 97 are scored) that measure different aspects of AEI: (A) Perceiving Emotions – children identify certain emotions in photographed facial expressions, (B) Using Emotions – children rank, using a standardized scale, the extent to which different emotions impact behavior and decision making, (C) Understanding Emotions – children read vignettes and select the answer representing what emotion the protagonist is feeling, (D) Managing Emotions – children read several scenarios and pick, from several options, the best solution for managing emotions in each scenario. Multi-Health Systems, the test distributor, scored the data using expert norms, providing means for each branch of the MSCEIT-Yv, and a total MSCEIT-Yv score. Acceptable split-half reliabilities have been obtained for the MSCEIT, e.g., 0.67 (perceiving) to 0.86 (understanding) and 0.90 for total AEI (Papadogiannis et al., 2009), and the hierarchical four-factor structure has been recovered in data from youth aged 9 to 15 years (Rivers et al., 2012). In this sample, branch scores were significantly intercorrelated [0.24 (correlation between Perceiving and Using branches)] to 0.70 (correlation between Understanding and Managing branches); See Table 1.

**Table 1:** Means and standard deviations with bivariate associations.

	Mean	SD	2.	3.	4.	5.	6.	7.	8.	9.	10.	11.
1. AEI perceiving emotion	85.62	13.27	0.24^**^	0.63^**^	0.52^**^	0.28^**^	-0.18^**^	-0.11	-0.11	-0.23^**^	-0.15^*^	-0.14^*^
2. AEI using emotion	104.11	10.71		0.34^**^	0.49^**^	-0.01	0.33^**^	0.35^**^	0.27^**^	0.20^**^	0.37^**^	0.29^**^
3. AEI understanding emotion	94.45	10.28			0.70^**^	0.15^*^	-0.13	-0.09	-0.04	-0.05	-0.10	-0.01
4. AEI managing emotion	96.95	8.79				0.25^**^	0.07	0.05	0.04	0.02	0.09	0.07
5. Global TEI	3.48	0.36					-0.33^**^	-0.24^**^	-0.13	-0.24^**^	-0.27^**^	-0.21^**^
6. Peer-related loneliness T1	28.79	8.54						0.80^**^	0.76^**^	0.75^**^	0.94^**^	0.80^**^
7. Peer-related loneliness T2	31.12	10.58							0.69^**^	0.71^**^	0.93^**^	0.72^**^
8. Depressive symptoms T1	5.57	3.86								0.76^*^	0.80^**^	0.91^**^
9. Depressive symptoms T2	6.48	4.42									0.81^**^	0.90^**^
10. Prolonged loneliness T1 × T2	978.55	559.05										0.87^**^
11. Prolonged depressive symptoms T1 × T2	57.77	69.54										


*^∗^*p* < 0.05, ^∗∗^*p* < 0.01, ^∗∗∗^*p* < 0.001; AEI, ability emotional intelligence (MSCEIT-Yv); TEI, trait emotional intelligence (TEIQue-CF).*

#### Trait Emotional Intelligence Questionnaire-Child Form (TEIQue-CF; Mavroveli et al., 2008)

The TEIQue-CF comprises 75 statements, tapping 9 facets of emotional functioning (e.g., emotion expression; emotion regulation; affective disposition). The child is asked to respond to each item using a 5-point Likert scale, ranging from “completely disagree” to “completely agree.” Example items include “I find it difficult to understand what others are feeling” and “If someone makes me angry, I tell them.” The TEIQue-CF has satisfactory levels of internal consistency for the global trait EI dimension (e.g., α = 0.89), but not at facet level (Russo et al., 2012; Stassart et al., 2017). Additionally, data collected with younger children suggest the tool is unidimensional in nature (Russo et al., 2012). In view of that evidence, current analyses were restricted to the use of the global TEI score (scored data provided by the test developers). In the current study, α = 0.87 for the full TEIQue-CF.

### Data Analysis

We hypothesized that enduring feelings of high loneliness and depression would be influenced by TEI and AEI. Duration scores for loneliness and depressive symptoms were computed by multiplying T1 and T2 scores (Qualter et al., 2010); higher scores were demonstrable of high, stable loneliness and high, stable depression over the 12 months of the study. Because we were interested in loneliness and depression scores that started high and stayed high over the 12 months of the study, we did not include the earlier T1 loneliness score in our regression model as a control variable.

To examine the effects of Global TEI and aspects of AEI on maintained loneliness and depressive symptoms, we ran two regressions, one with T1 × T2 loneliness as the DV, and the other with T1 × T2 depressive symptoms as the DV. In each regression, the following predictors were all included in one step: gender, MSCEIT-Yv subscales, and Global TEIQue-CF. Gender was dummy coded (gender: -1 = female, and +1 = male) as recommended by Cohen, Cohen et al. (2003); the MSCEIT-Yv subscales and Global TEIQue-CF variables were centered before inclusion in the regression analyses. We performed bootstrapping, estimating a 95% bias-corrected confidence interval for all values of interest, using 1000 bootstrap sample.

## Results

Means and standard deviations for each of measures are shown in Table 1, with bivariate associations between variables. Means show that the sample increased on loneliness and depressive symptoms from T1 to T2. Correlations suggest high stability of both loneliness and depressive symptoms over time. Loneliness and depressive symptoms were also related to one another within and over time. Loneliness at T1 and T2 was significantly negatively associated with the Perceiving Emotions branch of the MSCEIT-Yv, positively associated with Using Emotions, and negatively with the TEIQue-CF Global score. Depressive symptoms at T1 and T2 were positively associated with Using Emotions and at T2 higher reports of depressive symptoms were also negatively associated with scores on the Perceiving Emotions branch of the MSCEIT-Yv and the Global TEIQue-CF score.

Table 2 shows the bootstrap results from the regression analyses for prolonged depressive symptoms. There were no effects of gender on prolonged depressive symptoms, but Perceiving and Using Emotions branch scores of the MSCEIT-Yv predicted prolonged depressive symptoms. The positive beta for Using Emotions suggests that children in our sample who were skilled at using emotions to promote change in behavior were more likely than their peers to maintain depressive symptoms; the negative beta for Perceiving Emotions suggests that children in our sample who had difficulty reading the emotions of others were more likely than their peers to experience high levels of depressive symptoms over the 1 year of the study.

**Table 2:** Bootstrapped regression analysis with AEI and TEI as predictors of prolonged depressive symptoms.

Time 1 measures	B	SE B	*p*	95% CI
Constant	57.47	4.51		
Gender	4.94	4.50	0.272	-3.90, 14.09
**AEI**				
Perceiving emotions	-1.05	0.53	0.037	-2.20, -0.14
Using emotions	1.94	0.65	0.006	0.86, 3.40
Understanding emotions	-0.17	0.75	0.803	-1.65, 1.26
Managing emotions	0.71	1.03	0.465	-1.40, 2.71
**TEI**				
Global TEI	-30.67	18.30	0.090	-69.48, 2.09


*F = 6.53, *p* < 0.001, *R*^*2*^ = 0.16, Adjusted *R*^*2*^ = 0.14. AEI, ability emotional intelligence; TEI, trait emotional intelligence.*

Bootstrap results from the regression analyses for prolonged loneliness are noted in Table 3 and show that effects were moderated by gender. Follow-up analyses to examine those effects can be found in Table 4. For boys, higher scores on the Using Emotions branch of the MSCEIT-Yv and lower scores on the Understanding Emotions branch predicted the maintenance of peer-related loneliness. For girls, scoring higher on the Using Emotions and Managing Emotions MSCEIT-Yv branches and having a lower TEIQue-CF score predicted prolonged feelings of loneliness over the 12 months of the study.

**Table 3:** Bootstrapped regression analysis with AEI and TEI as predictors of prolonged loneliness.

Time 1 measures	B	SE B	*P*	95% CI
Constant	973.19	32.53		
Gender	87.74	33.71	0.009	20.97, 157.84
**AEI**				
Perceiving emotions	-4.97	4.32	0.244	-14.65, 2.47
Using emotions	19.92	5.01	0.001	11.14, 30.60
Understanding emotions	-15.35	5.89	0.010	-28.32, -4.01
Managing emotions	14.73	7.03	0.026	1.40, 29.11
**TEI**				
Global TEI	-350.98	145.33	0.016	-629.77, -52.29


*F = 14.26, *p* < 0.001, *R*^*2*^ = 0.29, Adjusted *R*^*2*^ = 0.27. AEI, ability emotional intelligence; TEI, trait emotional intelligence.*

**Table 4:** Bootstrapped regression analysis with AEI and TEI as predictors of prolonged loneliness for boys and girls.

Time 1 measures	Boys				Girls			
		
	B	SE B	*p*	CI	B	SE B	*p*	CI
Constant	1063.51	48.59			887.99	45.14		
**AEI**								
Perceiving emotions	-0.72	4.84	0.866	-9.94, 9.45	-10.20	8.02	0.195	-29.75, 0.90
Using emotions	21.97	7.93	0.007	9.44, 40.32	20.31	6.79	0.005	8.88, 35.19
Understanding Emotions	-20.74	7.37	0.006	-37.30, -8.04	-11.44	9.20	0.197	-32.35, 4.99
Managing emotions	10.12	13.50	0.428	-15.10, 39.50	19.05	8.36	0.026	4.85, 37.82
**TEI**								
Global TEI	-204.21	226.73	0.360	-686.12, 207.98	-503.08	199.68	0.013	-897.50, -116.04
	*F* = 9.38, *p* < 0.001, *R*^2^ = 0.31, Adjusted *R*^2^ = 0.27				*F* = 7.65, *p* < 0.001, *R*^2^ = 0.29, Adjusted *R*^2^ = 0.25			


*AEI, ability emotional intelligence; TEI, trait emotional intelligence.*

For prolonged loneliness, we explored whether global TEI acted as a suppressor variable in the regression. We explored suppression effects given that (a) the MSCEIT-Yv understanding and managing emotion subscale scores were uncorrelated with loneliness at T1, T2, and prolonged loneliness (T1 × T2), but were significant predictors in the regression models for boys and girls, respectively, and (b) global TEI was correlated with both branches and T1 and T2 loneliness, and prolonged loneliness, but was not significant in the regression for boys. We explored whether global TEI provided suppression by determining whether there was an increase in beta weights for understanding and managing emotions when global TEI was included in the regression model. Such an exploration is important for understanding the relationships between variables in our dataset. Bootstrapping results (see Supplementary Materials I) show that for girls, the inclusion of global TEI increased the standardized beta weight for MSCEIT-Yv Managing scores, so that it was now a significant predictor (*B* moved from 11.16 to 19.05, *p* moved from 0.178 to 0.018); for boys, the addition of global TEI, while not a significant predictor of prolonged loneliness, unsuppressed the underlying pattern so that now MSCEIT-Yv Understanding scores were more strongly related to prolonged loneliness (*B* moved from -19.24 to -20.74, *p* moved from 0.014 to 0.010) than when TEI was not included in the model.

## Discussion

In the current study, we explored the independent roles of emotional skills (AEI) and emotional self-competency (TEI) in the maintenance of depressive symptoms and loneliness in school children aged 9—11 years. This is the first examination of those longitudinal associations in middle childhood. Our findings showed that emotional skills are important for predicting the maintenance of depressive symptoms and feelings of loneliness during childhood, whereas emotional self-competency acted more selectively; TEI did not predict the maintenance of depression and predicted enduring loneliness for girls only. Those findings conflict with existing youth-focused cross-sectional literature that has found TEI, not AEI, predictive of a broader range of disorders (Williams et al., 2009; Davis and Humphrey, 2012). Our findings also suggest that emotional skills play different roles in the maintenance of internalizing symptoms, and that universally “high” levels may not always be adaptive. Deficiencies in perceiving and understanding emotions were predictive of prolonged symptomatology, yet so, too, were *high* levels of emotion management skill and ability to use emotion to facilitate thinking. Such findings, discussed further below, have important implications for the theory and study of emotional intelligence, and the design of interventions that aim to teach children to perceive, understand, use, and manage emotions.

### Emotional Intelligence and Internalizing Symptoms

In our sample of children, superior ability to “use” emotional information contributed to both the maintenance of depressive symptoms and loneliness. Although that predictive pattern appears at odds with AEI theory (i.e., that higher skills should relate to better wellbeing; Mayer et al., 2008), scrutiny of the MSCEIT-Yv “Using Emotion” test items helps to clarify why this might be important for the maintenance of internalizing disorder over time. The adult MSCEIT taps ability to use emotion to influence thinking (e.g., knowing which emotions might be useful for creativity, problem-solving or how changing thoughts can be useful for improving mood), but the youth test items provide an index of children’s conscious *awareness of emotional experience*. Items require children to compare their experience of specific feelings (e.g., liking someone) to different sensations, such as color, temperature, or speed (e.g., warm, soft, pink etc.). In order to do that task successfully, children need to have experienced the feelings and be able to recognize individual emotions when they occur. Such a skill, then, requires attending to feelings and monitoring emotions, which may not always be adaptive. Experiencing heightened emotional intensity (both positive and negative emotions) and increasing one’s attention to moods are both associated with internalizing disorders (Mennin et al., 2007; Suveg et al., 2009; Lizeretti et al., 2012; Boden and Thompson, 2015). Indeed, it has been suggested that an inability to shift attention away from (negative) emotions may precipitate disorder through ruminative thinking (Joormann and Gotlib, 2008).

In order to fully understand why having heightened emotional insight is disadvantageous, we need to consider the combined “profile” of skills and perceived competencies that significantly predicted the maintenance of depression and loneliness in the current sample of children. That is important because individuals could possess a “vulnerable” EI profile, where imbalances in the levels of constituent EI skills or trait-level facets (e.g., emotionally perceptive/poor regulators) predict poorer intrapersonal outcomes (Davis and Nichols, 2016). In the context of internalizing disorders, high levels of emotional awareness have been linked to use of adaptive and maladaptive emotion regulation strategies in children, adolescents, and adults (Suveg et al., 2009; Eastabrook et al., 2014; Boden and Thompson, 2015), suggesting that by experiencing emotions, individuals develop an understanding of the suitability and efficacy of different management strategies for specific environmental contexts. Put simply, if children are not aware of their own emotions, they cannot build an understanding of how to regulate them effectively. Our data supports that pattern: girls who are highly skilled in using (feeling) emotion also had good knowledge of how to manage emotion in themselves and others. However, those girls also maintained their feelings of loneliness over 1 year. That can be qualified with reference to perceived emotional competency (TEI): those girls in our study with high levels of emotional skill also reported that they lacked confidence in their competencies. Without the confidence to enter the social arena to deploy their emotional knowledge, establishing meaningful social relationships may be difficult, maintaining feelings of loneliness. The interdependence of skills and self-efficacies in the prediction of internalizing disorder is an emerging theme in the EI literature that has been demonstrated elsewhere in cross-sectional work with adolescents (Davis and Humphrey, 2014) and female adults (Salguero et al., 2015). The current findings support the idea that without average-high levels of TEI, high levels of skill are redundant.

Interestingly, in our sample of boys, TEI was not important for predicting the maintenance of loneliness over time. For boys, having high levels of emotional awareness (using emotion) coupled with poor emotional understanding was influential. The MSCEIT-Yv measures knowledge of emotion vocabulary and complex emotional blends (e.g., the constituent components of calmness). It is possible, therefore, that boys who are good at recognizing the sensation of emotions they experience, but are unable to label those complex emotions and understand how they transition from more basic feelings, continue to experience loneliness. Without sufficient knowledge, those boys may be unsure how emotional interactions will play out – they are poorer at communication, yet feel their social disconnection acutely.

Contrary to previous literature (e.g., Gomez-Baya et al., 2017), we found no gender differences in the maintenance of depression symptoms. Across our sample of children, being skilled at feeling (using) emotion, but poor at identifying emotions in others, predicted long-term internalizing problems, measured as enduring depressive symptoms and loneliness. Biases in the processing of emotional expressions are well established in the literature, with studies typically finding that depressed young people and adults perceive positive, negative, and ambiguous emotions as more negatively valenced, particularly those presented at lower intensities (Bourke et al., 2010; Schepman et al., 2012); loneliness is also related to the attribution of negative intentions to ambiguous social encounters (Qualter et al., 2013; van Roekel et al., 2015) and cognitive biases for negative affect (Qualter et al., 2013, 2015b; Bangee et al., 2014; Spithoven et al., 2017). Since successful interaction depends upon being able to accurately identify non-verbal emotional signals in others, continued misattribution of emotional signals (e.g., perceiving individuals as less happy) could prolong negative mood states in children and lead to further socio-emotional problems.

It is notable that we did not find a predictive effect of TEI in the maintenance of depressive symptoms in our sample because previous cross-sectional studies have shown a link (Williams et al., 2009; Davis and Humphrey, 2012). However, the age range of our sample necessitated the use of the TEIQue child form, which differs substantially in content coverage from the adolescent version, used in the related previous studies. The TEIQue-CF measures a complex blend of perceived emotional competencies (e.g., emotion expression), facets of emotional personality (e.g., affective disposition) and self-beliefs (e.g., motivation) (Mavroveli et al., 2008). The newly developed Emotional Self-Efficacy Scale (ESE; Qualter et al., 2015a) offers a “cleaner” examination of discrete perceived emotional competencies in children, and as a next step it would be desirable for researchers to validate and extend the current findings using this tool.

### Strengths, Limitations and Future Directions for Research

The current study provides an examination of the association between emotional intelligence and the maintenance of depressive symptoms and loneliness over 1 year in children. Given that much of the previous work has been with older adults and older adolescents and has focused on cross-sectional influences, we have filled a knowledge gap, providing data on how AEI and TEI influence the maintenance of depressive symptoms and feelings of loneliness. The longitudinal design is a strength of the study, enabling the examination of how emotion skills and self-competencies impact the maintenance of depression and loneliness. However, data were collected over a relatively short period of childhood. Although this is an important first step for confirming relationships at an early stage of development, it will be important to extend this work to track symptomatology across a longer time period into adolescence, where we would expect normative increases in depression and loneliness (Laursen and Hartl, 2013; Wong et al., 2018). This is also important from a replication perspective, to establish that our results were not due to idiosyncratic cohort effects.

Researchers may also consider refining the tools used to capture those relationships. Specifically, the use of an age-appropriate TEI measure that accommodates sub-facet analysis (e.g., ESE: Qualter et al., 2015a) would allow more fine-grained examination of how competency beliefs about specific emotion skills (e.g., confidence in regulating one’s own emotions/the emotions of others etc.) impact wellbeing across time. Use of a TEI measure that includes subscales, and, thus, narrower bandwidths, would mean that the importance of lower level TEI facets could be examined alongside lower level AEI dimensions, offering greater predictive precision due to increased instrument fidelity (e.g., Ones and Viswesvaran, 1996; Paunonen and Ashton, 2001). In future work, it will be important for any comparison between EI measures to consider the level at which each test is assessed, and make them as comparable as possible. Additionally, it will also be important to examine the incremental validity of both AEI and TEI to predict prolonged symptoms beyond the influence of allied constructs (e.g., broadband personality dimensions and general cognitive ability) that are known predictors of wellbeing and/or EI. In adolescents, there is evidence that both AEI and TEI can explain unique variance in mental wellbeing (Davis and Humphrey, 2012; Frederickson et al., 2012; Rivers et al., 2012), but that has not been explored in children.

### Implications for Theory and Practice

Our findings confirm the value of studying discrete groups (e.g., girls, boys) and EI profiles to establish patterns of specificity and commonality in the maintenance of internalizing disorders. These data clearly contrast with the theoretical notion that EI is universally beneficial for adaptive outcomes (e.g., Mayer et al., 2008), and suggest that EI theory should be modified and extended to incorporate contextual effects, akin to developmental theories of emotional competence (e.g., Saarni, 1999). Our findings also emphasize the importance of studying longitudinal links between both types of EI and any mental health problem: the current findings differ in important ways from cross-sectional data (e.g., compared with AEI, TEI is a weaker determinant of prolonged vs. concurrent disorder) and underscore the divergent roles played by perceptions of emotional competencies vs. actual abilities. It is notable that through our examination of the combined “EI profile” of skill and self-competency, we detected classical suppression effects in our loneliness data. Statistically, the inclusion of TEI in predictive models “purified” the direction of predictive effects of emotion skills (understanding, management, using) on prolonged loneliness. Whilst this could indicate that TEI and AEI share some common methodological elements that are irrelevant in the prediction of prolonged loneliness (e.g., measurement error), theoretically, this further underscores the importance of considering TEI and AEI as a *composite* predictor of adjustment outcomes (i.e., how important self-confidence in skills is for enabling abilities). By including TEI and AEI together in predictive models, researchers can better understand how social and emotional skills and self-efficacies can be trained in an optimal manner. Replication of our effects is now required to build consensus in the literature and develop clear guidelines for intervention design.

Our finding that global TEI was less predictive of enduring depression and loneliness than individual AEI subscales could be seen to challenge previous work suggesting measurement reliability is maximized when global EI constructs are examined because there is broader bandwidth (Gardner and Qualter, 2010). Broader bandwidth can result in the prediction of a wider range of criteria (Ones and Viswesvaran, 1996), but in our study that was not the case: we found that global TEI score predicted less variance in enduring depressive symptoms and prolonged loneliness than subscale AEI scores across genders, suggesting a robust effect. Of course, one could argue that because global trait EI carries only one degree of freedom and the four AEI factors carry four degrees of freedom and are measured at similar bandwidths, comparison between global TEI and AEI subscales is non-equivalent. Thus, in future work, researchers will want to choose EI measures that can be treated similarly because they have comparable bandwidth, extending our understanding of the incremental validity of those tests to predict the maintenance of internalizing symptoms.

Our findings also have implications for the teaching of emotional information. Social and emotional learning interventions (SEL) aim to develop social and emotional skills through explicit instruction in schools (Humphrey, 2013) and there is evidence that universal (i.e., whole school) programs are effective in training socio-emotional competencies to improve wellbeing (Durlak et al., 2011; Sklad et al., 2012; Wigelsworth et al., 2016). Our finding that deficiencies in emotion perception, understanding, and emotion self-efficacies differentially predict the maintenance of depression and loneliness supports the theory of change that underpins SEL (CASEL, 2007), aligning well with the findings that SEL interventions promote effective coping and social engagement (Goodman et al., 2015). However, contrary to that movement, we found that some skills (e.g., feeling and emotion management knowledge) were not necessarily protective against prolonged internalizing symptoms and that this differed for girls and boys. Those findings suggest that to be most effective, SEL programs need to include a tiered system, where the needs of specific groups of “at risk” children can be addressed (Humphrey et al., 2016).

## Conclusion

The current study explored the role of emotional knowledge, measured within the AEI and TEI frameworks, in the maintenance of depressive symptoms and loneliness in children aged 9–11 years. We found that deficiencies in the ability to perceive and understand emotions predicted the maintenance of symptomatology, but proficiencies in using emotion to facilitate thinking and emotion management were also important. Those findings have implications for EI theory and future research, and for intervention work designed to build emotional knowledge and/or reduce internalizing problems. First, there is a need to examine the combined profiles of AEI and TEI in future work with children and adolescents because it seems that having emotional skills is redundant if the child does not have the confidence to use those skills. Second, a modification to AEI theory is needed to incorporate contextual effects; our findings suggest that the utility of AEI skills cannot be determined without reference to other qualities of the person (e.g., gender; levels of perceived emotional competency), but also the nature of behavioral outcomes studied. The maintenance of loneliness in the young girl who is emotionally skilled, but lacking in emotional confidence, may be precipitated by challenging situational factors (e.g., difficulty re-connecting with peers) and she may feel emotional setbacks acutely, perpetuating this cycle. There is a need to move beyond this narrow-band, static approach to AEI. Third, while there is an argument for universal interventions designed to build emotional knowledge among children and adolescents, we argued a tiered system that allows tailored interventions for groups of “at risk” children may also be useful for reducing specific profiles of internalizing symptoms.

## Data Availability

The raw data supporting the conclusions of this manuscript will be made available by the authors, without undue reservation, to any qualified researcher.

## Ethics Statement

Details of our ethical consent procedure can be found in the methods section of the manuscript. We can confirm that parental consent was provided by parents at both waves of data collection and child participants provided assent during data collection. This study was carried out in accordance with the recommendations of the ethics panel at The University of Central Lancashire, who approved the study.

## Author Contributions

All authors conceived the study. SD participated in the study design, interpreted the findings, wrote the initial manuscript, and made revisions to the manuscript based on reviewers’ feedback. PQ performed statistical analyses, interpreted the findings, and wrote the initial manuscript. RN helped to perform statistical analyses, interpret the findings, and contributed to the draft manuscript. PQ and RN coordinated the original data collection. All authors read and approved the final version of the manuscript.

## Conflict of Interest Statement

The authors declare that the research was conducted in the absence of any commercial or financial relationships that could be construed as a potential conflict of interest. The reviewer KG declared a shared affiliation, with no collaboration, with one of the authors, RN, to the handling Editor at the time of review.
