# Supplementary material for: The Role of Emotional Intelligence in the Maintenance of Depression Symptoms and Loneliness Among Children
Source: Front Psychol. 2019 Jul 17;10:1672. doi: 10.3389/fpsyg.2019.01672 (PMC6660264; doi:10.3389/fpsyg.2019.01672)
Supplement: Supplementary file 1 [file Data_Sheet_1.pdf]

The Role of Emotional Intelligence in the Maintenance of Depression Symptoms and Loneliness Among Children

https://www.frontiersin.org/articles/10.3389/fpsyg.2019.01672/full#supplementary-material

Supplementary Materials I

Table S1. Bootstrapped Step-wise Regression Analysis with AEI and TEI as predictors of Prolonged Loneliness for Boys and Girls.

Time 1 Measures	Boys				Girls			
	B	SE B	*P*	CI	B	SE B	*p*	CI
**Step 1**								
Constant	-93.96	671.11	.883	-1533.22, 1129.74	-698.30	697.55	.307	-2175.92, 681.05
AEI Perceiving Emotions	-2.76	4.72	.543	-13.31, 6.10	-11.96	9.42	.223	-33.19, 1.81
AEI Using emotions	23.53	7.60	.003	11.61, 40.09	21.38	7.33	.011	9.90, 38.13
AEI Understanding Emotions	-19.24	7.60	.014	-35.15, -3.54	-7.55	8.58	.351	-26.53, 6.04
AEI Managing Emotions	7.92	13.42	.550	-19.25, 33.311	11.16	8.97	.178	-4.26, 31.10
	F = 11.18, $p < .001$, $R^2 = .29$, Adjusted $R^2 = .27$				F = 5.19, $p < .001$, $R^2 = .18$, Adjusted $R^2 = .15$			

Step 2								
Constant	525.18	1011.83	.589	-1642.97, 2385.58	628.47	947.49	.525	-1329.06, 2406.76
AEI Perceiving Emotions	-.72	4.84	.856	-10.20, 10.52	-10.20	7.93	.180	-28.98, .76
AEI Using emotions	21.97	7.89	.007	9.84, 40.24	20.31	7.07	.008	8.61, 36.73
AEI Understanding Emotions	-20.74	7.38	.010	-36.45, -6.75	-11.44	8.99	.184	-30.91, 4.25
AEI Managing Emotions	10.12	13.30	.450	-17.24, 36.20	19.05	8.28	.018	3.77, 36.70
Global TEI	-204.21	213.85	.361	-656.72, 177.53	-503.08	194.24	.012	-884.93, -122.87
	F = 9.38, $p < .001$, $R^2 = .31$, Adjusted $R^2 = .27$				F = 7.65, $p < .001$, $R^2 = .29$, Adjusted $R^2 = .25$			

Notes: AEI = Ability Emotional Intelligence; TEI = Trait Emotional Intelligence
